# Pre-pregnancy BMI-specific optimal gestational weight gain for women in Japan

**DOI:** 10.1016/j.je.2016.09.013

**Published:** 2017-05-31

**Authors:** Naho Morisaki, Chie Nagata, Seung Chik Jwa, Haruhiko Sago, Shigeru Saito, Emily Oken, Takeo Fujiwara

**Affiliations:** a Department of Social Medicine, National Center for Child Health and Development, Tokyo, Japan; b Division of Education for Clinical Research, National Center for Child Health and Development, Tokyo, Japan; c Center of Maternal-Fetal, Neonatal and Reproductive Medicine, National Center for Child Health and Development, Tokyo, Japan; d Department of Obstetrics and Gynecology, University of Toyama, School of Medicine, Toyama, Japan; e Department of Population Medicine, Harvard Medical School, Harvard Pilgrim Health Care Institute, Boston, MA, USA

**Keywords:** Gestational weight gain, Body mass index, Japanese, Pregnancy

## Abstract

**Background:**

The Institute of Medicine (IOM) guidelines are the most widely used guidelines on gestational weight gain; however, accumulation of evidence that body composition in Asians differs from other races has brought concern regarding whether their direct application is appropriate. We aimed to study to what extent optimal gestational weight gain among women in Japan differs by pre-pregnancy body mass index (BMI) and to compare estimated optimal gestational weight gain to current Japanese and Institute of Medicine (IOM) recommendations.

**Methods:**

We retrospectively studied 104,070 singleton pregnancies among nulliparous women in 2005–2011 using the Japanese national perinatal network database. In five pre-pregnancy BMI sub-groups (17.0–18.4, 18.5–19.9, 20–22.9, 23–24.9, and 25–27.4 kg/m^2^), we estimated the association of the rate of gestational weight gain with pregnancy outcomes (fetal growth, preterm delivery, and delivery complications) using multivariate regression.

**Results:**

Weight gain rate associated with the lowest risk of adverse outcomes decreased with increasing BMI (12.2 kg, 10.9 kg, 9.9 kg, 7.7 kg, and 4.3 kg/40 weeks) for the five BMI categories as described above, respectively. Current Japanese guidelines were lower than optimal gains, with the lowest risk of adverse outcomes for women with BMI below 18.5 kg/m^2^, and current IOM recommendations were higher than optimal gains for women with BMI over 23 kg/m^2^.

**Conclusion:**

Optimal weight gain during pregnancy varies largely by pre-pregnancy BMI, and defining those with BMI over 23 kg/m^2^ as overweight, as proposed by the World Health Organization, may be useful when applying current IOM recommendations to Japanese guidelines.

## Highlights

• We estimated gestational weight gain that optimizes pregnancy outcomes in Japanese.
• Optimal gain was 12.2 kg/40 weeks for pre-pregnancy BMI 17.0–18.4 kg/m^2^.
• Optimal gain was 4.3 kg/40 weeks for pre-pregnancy BMI 25.0–27.4 kg/m^2^.
• Current national guidelines are too low for women with pre-pregnancy BMI < 18.5 kg/m^2^.
• BMI > 23 kg/m^2^ should be defined as overweight if applying IOM recommendations.

## Introduction

To our knowledge, there is no global consensus on the recommended amount of gestational weight gain. The Institute of Medicine (IOM) guidelines^1^ (last revised in 2009) are the most widely used in the world, although they were initially created only for use in the United States. As substantial evidence was included in creating and revising the IOM guidelines, the majority of countries that have national recommendations^2^, ^3^ have adapted these guidelines, and research studies based in other countries often refer to them in studies.^4^, ^5^, ^6^, ^7^

However, as the guidelines were based mainly on research in Caucasians, its generalizability to other races has been a concern among countries with a large population of non-European ancestry. Recent research show many Asian populations are at an increased risk of cardiovascular diseases and diabetes at lower BMI levels than non-Asians,^8^ leading to the World Health Organization (WHO) proposal in 2004 to use a “modified” threshold of BMI over 23 kg/m^2^ rather than the customary 25 kg/m^2^ to define overweight condition for Asians.^9^ In 2014, the American Diabetes Association decided to lower the BMI threshold for diabetic screening among Asians from 25 kg/m^2^ to 23 kg/m^2^, based on multiple studies that implied that a lower threshold would increase sensitivity.^10^, ^11^, ^12^, ^13^ Such different body composition in Asians could also modify the influence of gestational weight gain on pregnancy outcomes. Following the WHO expert panel proposal, New Zealand^2^, ^3^ and Singapore^14^ considered adopting the modified BMI thresholds for Asians upon applying the IOM recommendation of gestational weight gain to mothers of Asian origin (and thus lowering recommended weight gain for Asian women of BMI 23–25 kg/m^2^); however, due to the lack of studies on gestational weight gain and BMI thresholds in Asians, both countries decided to adapt the recommendations without modification.

On the other hand, Japan has not adopted the IOM guidelines but developed and adhered to original guidelines, first published in 1995 and revised in 2009. These guidelines have been considerably stricter than IOM guidelines, and recent debate in Japan has questioned whether such a strict guideline has contributed to the increasing rate of low-birthweight births in the country.^15^ One large limitation of the Japanese guidelines is that they lack evidence from a large study in Japan that has looked at the effect of weight gain during pregnancy by simultaneously considering multiple outcomes, as has been done in the United States,^16^ Denmark,^17^ Singapore,^18^ and elsewhere.^19^

Therefore, using a national multi-center database of obstetric departments in Japan, we investigated how optimal gestational weight gain varies depending on pre-pregnancy BMI in Japanese women and compared estimated optimal gestational weight gain to current Japanese and Institute of Medicine (IOM) recommendations.

## Methods

### Study population

We used data from the perinatal database collected by the Japan Society of Obstetrics and Gynaecology (JSOG), an ongoing multi-center registry for all deliveries, in which 149 tertiary hospitals in Japan are currently participating.^20^, ^21^, ^22^, ^23^, ^24^, ^25^, ^26^ This database is a national collaboration of obstetric departments that started in 2001. Using a standardized format that includes demographic and clinical variables, each hospital provided individual patient data on all live births and stillbirths that were delivered at 22 weeks or above. The JSOG Perinatal Committee reviewed the submitted data for data quality and contacted institutes for correction if a substantial portion contained erroneous or missing data. The data for 2004–2011 was recently linked to vital statistics data that verified its accuracy and showed that this registry covered 6.5% of all national births.

As both gestational weight gain and pregnancy outcomes are affected by underlying medical conditions, and as our database lacked data on previous cesarean delivery, which is a large risk factor of cesarean delivery, we restricted our analysis to 118,878 nulliparous women with pre-pregnancy BMI of 17.0 kg/m^2^ to 27.5 kg/m^2^ (the number of women in more extreme BMI categories [BMI ≤16 kg/m^2^, *n* = 832; BMI 16–16.9 kg/m^2^, *n* = 3713; BMI 27.5–30 kg/m^2^, *n* = 1860; and BMI 30–35 kg/m^2^, *n* = 1524] were too small to calculate stable estimates of the outcomes of interest), and who had no medical conditions (any chronic respiratory, gastrointestinal, hepatic, renal, hematologic, cardiovascular, metabolic, mental diseases, pre-gestational hypertension or diabetes mellitus) before or during early pregnancy, did not have gestational diabetes (as our database could not differentiate diabetes mellitus from gestational diabetes), and gave birth to singletons between April 2005 and December 2011. The number of infants with congenital anomalies documented at birth was extremely low (<0.1%), suggesting substantial underreporting, and thus were not excluded. Women with unreliable reports of gestational weight gain (outside ±3 SD above mean, equivalent to >23.3 kg or <−1.9 kg, *n* = 1629) and those with missing gestational weight gain data (*n* = 10,431) were also excluded. We further excluded extremely preterm deliveries (GA < 28 weeks, *n* = 511) or participants with missing data on gestational age, maternal age, or delivery method (*n* = 1539), and those with missing or unreliable combinations^27^ of birthweight and gestational age (*n* = 309). All remaining participants had data on all variables of interest, so our total sample for analysis included 104,070 women.

### Expected gestational weight gain over 40 weeks

One important limitation of previous studies is the management of gestational weight gain in their analyses. As gestational weight gain relies largely on gestational length, studying the effect of total gestational weight gain, as in many previous studies,^5^, ^6^, ^17^, ^28^, ^29^ skews results by creating a false association between birth outcomes related to shorter gestational length and lower gestational weight gain. For example, the mere fact that women with preterm deliveries have less weight gain is not informative. The true comparison for studying the effect of weight gain on preterm delivery should be based on “rate” of weight gain, or how much women with preterm delivery would have gained if they continued to gain weight until term. An alternative method is to obtain the weekly rate of weight gain by dividing total gain by number of gestational weeks^16^; however, this method is based on an underlying assumption that weight gain is linear throughout pregnancy, which has been reported to be untrue, as gestational weight gain speed turns linear only after the first 3 or 4 months.^8^

To overcome these methodological challenges, we created a model using data from a subsample of 1283 mothers, including their detailed measurements from antenatal check-ups, to calculate expected gestational weight gain over 40 weeks from clinical measurement on admission for delivery and self-reported pre-pregnancy weight, and used this for the main analysis (details on analysis in eAppendix 1). This methodology enabled us to compare the effect of gestational weight gain between women who delivered at different gestational ages and to deal with perinatal outcomes, such as preterm delivery.

### Birth outcomes

We considered a variety of pregnancy outcomes, both individually as well as combined, and weighted them by clinical importance. Gestational length was based on best obstetric estimate, which is estimated from the last menstrual period (LMP) and corrected according to ultrasound measurements. In detail, JSOG recommends LMP estimates of gestational age to be corrected according to ultrasound measurements of crown-lump (CRL) length if before 12 weeks gestation and ultrasound and LMP estimates differ by over 7 days, or corrected according to ultrasound measurements of bi-parietal diameter if CRL measurements were not available in the first trimester and ultrasound and LMP estimates differ by over 10 days. Infant death was determined from the linked vital statistics database. Preterm birth was defined as less than 37 completed weeks of gestation, and very preterm birth was defined as less than 34 completed weeks of gestation. Obstructed labor was defined as labor of over 30 h, and post-partum hemorrhage was defined as blood loss of over 500 mL. We additionally defined a composite outcome of obstructed labor, post-partum hemorrhage, delivery using forceps and vacuum, or cesarean delivery (labeled as ‘complicated delivery’).

JSOG defines preeclampsia as elevated blood pressure ≥ 140/90 mm Hg that emerges after 20 weeks' gestation with proteinuria, and severe preeclampsia if systolic blood pressure exceeds 160 mm Hg, diastolic blood pressure exceeds 110 mm Hg, or proteinuria exceeds 2 g/day.^30^ Clinically, preeclampsia may cause edema, leading to an increase in weight gain; therefore, we conducted sensitivity analysis both including and excluding preeclampsia from the primary outcome.

Fetal size was defined as small for gestational age (SGA) and large for gestational age (LGA) as below 10% or above 90%, respectively, of the birthweight distribution by gestational length of all births in Japan during the study period. However, as the average birthweight in Japan has decreased dramatically over the last 30 years, and the current birthweight distribution could have deviated from that of a healthy population,^31^ we also repeated our analysis using the WHO reference to define fetal size. Low birthweight, very low birthweight, and macrosomia were defined as birthweight <2500 g, <1500 g, and ≥4000 g, respectively.

Upon estimating optimal gestational weight gain, we considered the trade-off between multiple outcomes, and for this, we used weights to account for the fact that certain outcomes have more clinical importance than others.^16^ To calculate these weights, we asked 12 neonatologists and 15 obstetricians at the National Center for Child Health and Development to rank SGA, preterm delivery, complicated delivery, and preeclampsia in order of their adverse health effects, as well as how many times worse the most adverse outcomes were compared to the least adverse outcome, and averaged these answers. Obstetricians selected SGA as the least adverse, with preterm delivery and preeclampsia being 2.3 times and complicated delivery being 1.2 times more adverse than SGA. Neonatologists also selected SGA as the least adverse, with preterm delivery being 1.4 times, preeclampsia being 1.2 times, and complicated delivery being 1.5 times more adverse than SGA. In total, the clinicians selected SGA as the least adverse, with preterm delivery being 1.8 times, preeclampsia being 1.7 times, and complicated delivery being 1.4 times more adverse than SGA.

### Statistical analysis

We calculated pre-pregnancy BMI from self-reported height and pre-pregnancy weight, and stratified women accordingly into five groups: BMI 17.0–18.4, 18.5–19.9, 20–22.9, 23–24.9, and 25–27.4 kg/m^2^. Further analyses were performed within these groups.

First, within each BMI category, we performed a multivariate logistic regression predicting all outcomes of interest according to expected gestational weight gain. For these models, expected gestational weight gain was categorized by each kilogram and used as a non-parametric model to avoid forcing the shape of the association. For fetal size (SGA, appropriate for gestational age, LGA) and birthweight (<2500 g, 2500–3999 g, ≥4000 g), multinomial logistic regression was used. All models were adjusted for maternal age, height, pre-pregnancy BMI, and smoking status during pregnancy.

Next, we chose preterm delivery, SGA, complicated delivery, and preeclampsia as the four clinically important outcomes for which risks are known to be affected by gestational weight gain. For these four main outcomes of interest, another set of multivariate logistic models were run concurrently using weight gain rate as a continuous variable to correct for correlation among outcomes within each individual. From these models, the expected gestational weight gain at 40 weeks (calculated from rate of weight gain) with lowest-risk of adverse outcome was estimated for each BMI category. The range of expected gestational weight gain within 1% increased risk of adverse outcome was defined as the acceptable range of gestational weight gain rate. A quadratic term was included to improve the fitness of the model.

For sensitivity analysis, we repeated these models using the WHO growth standard to define SGA instead of the Japanese growth standard. Although prevention of preeclampsia has historically been an important factor in gestational weight gain recommendations in Japan,^15^, ^32^ in 2008 the IOM working group decided^1^ there is only weak evidence that excessive weight gain could cause preeclampsia,^33^ as the observed association is likely due to reverse causation (edema due to preeclampsia causing increased weight gain). Based on this decision, the IOM working group has eliminated preeclampsia from the main outcomes when determining optimal weight gain.^1^ Thus, we repeated our analysis excluding this outcome.

All descriptive and statistical analyses were performed using STATA version 13 (STATA Corp, College Station, TX, USA). Statistical significance was set at 0.05, and all statistical tests were two-tailed.

## Results

Among our sample population, 100,772 (96.8%) women were Japanese, 438 (0.4%) were Korean, 1417 (1.4%) were Chinese, and 1443 were of other nationalities.

In Table 1, we present maternal and infant characteristics according to pre-pregnancy BMI categories. Women with higher BMI were older and shorter and had less gestational weight gain. The prevalence of cesarean delivery, forceps or vacuum delivery, obstructed labor, post-partum hemorrhage, and preeclampsia all increased with higher pre-pregnancy BMI. Similarly, prevalence of LGA, infant asphyxia, and infant mortality also increased with higher pre-pregnancy BMI. Prevalence of SGA and early-term delivery decreased with increasing pre-pregnancy BMI.

**Table 1:** Background characteristics of 104,070 singleton pregnancies in nulliparous Japanese women.

	BMI 17–18.4 kg/m^2^	BMI 18.5–19.9 kg/m^2^	BMI 20–22.9 kg/m^2^	BMI 23–24.9 kg/m^2^	BMI 25–27.4 kg/m^2^	*P* value for trend
*N* (%)	19,311 (19)	32,099 (31)	39,636 (38)	8759 (8)	4265 (4)	

Maternal age, years, mean (SD)	29.7 (5.0)	30.2 (5.0)	30.4 (5.2)	30.7 (5.4)	30.7 (5.5)	<0.001
Maternal height, cm, mean (SD)	158.8 (5.4)	158.7 (5.4)	158.1 (5.4)	157.8 (5.5)	157.8 (5.4)	<0.001
BMI, kg/m^2^, mean (SD)	17.9 (0.4)	19.3 (0.4)	21.2 (0.8)	23.8 (0.6)	26.1 (0.7)	<0.001
Maternal smoking, *n* (%)						
Yes	573 (3)	858 (3)	1097 (3)	304 (4)	190 (5)	<0.001^a^
No	12,953 (67)	21,528 (67)	26,344 (67)	5824 (67)	2829 (66)	
Unknown	5785 (30)	9713 (30)	12,195 (31)	2631 (30)	1246 (29)	0.42^a^
Gestational weight gain rate, kg/40 weeks, mean (SD)	11.0 (3.5)	10.9 (3.5)	10.8 (3.8)	10.3 (4.4)	9.5 (4.6)	<0.001
Gestational age, weeks, mean (SD)	38.6 (2.0)	38.7 (2.0)	38.7 (2.1)	38.6 (2.2)	38.6 (2.2)	<0.001
Infant sex, *n* (%)						0.15
Male	9891 (51)	16,503 (51)	20,573 (52)	4460 (52)	2250 (51)	
Female	9420 (49)	15,596 (49)	19,063 (48)	4299 (49)	2015 (47)	
Birthweight, g, mean (SD)	2827 (479)	2881 (481)	2922 (508)	2947 (537)	2966 (552)	<0.001
Fetal size, *n* (%)						
Small for gestational age	3048 (16)	4216 (13)	4458 (11)	890 (10)	436 (10)	<0.001^a^
Appropriate for gestational age	15,115 (78)	25,352 (79)	31,131 (79)	6749 (77)	3193 (75)	
Large for gestational age	1148 (6)	2531 (8)	4047 (10)	1120 (13)	636 (15)	<0.001^a^
Delivery method, *n* (%)						
Normal vaginal delivery	13,877 (72)	22,509 (70)	26,556 (67)	5417 (62)	2539 (60)	
Elective cesarean section	1396 (7.2)	2412 (7.5)	3156 (8.0)	793 (9.1)	381 (8.9)	<0.001^a^
Emergency cesarean section	2240 (12)	4119 (13)	6089 (15)	1685 (19)	948 (22)	<0.001^a^
Forceps or vacuum delivery	1798 (9.3)	3059 (9.5)	3835 (9.7)	864 (9.9)	397 (9.3)	<0.001^a^
Preeclampsia, *n* (%)	694 (4)	1228 (4)	2126 (5)	715 (8)	435 (10)	<0.001
Obstructed labor, *n* (%)	929 (5)	1891 (6)	2659 (7)	676 (8)	335 (8)	<0.001
Atonic bleeding, *n* (%)	788 (4.1)	1364 (4.2)	1815 (4.6)	443 (5.1)	247 (5.8)	<0.001
Apgar score <8 at 5 min, *n* (%)	489 (2.6)	881 (2.8)	1148 (2.9)	326 (3.8)	163 (3.9)	<0.001
Gestational age, *n* (%)						
Preterm (<37 weeks)	2104 (10.9)	3178 (9.9)	4016 (10.1)	951 (10.9)	479 (11.2)	0.28^a^
Early term (37 or 38 weeks)	2104 (27)	3178 (26)	4016 (25)	951 (25)	479 (24)	<0.001^a^
Term/over term delivery (≥39 weeks)	11,958 (62)	20,596 (64)	25,886 (65)	5633 (64)	2759 (65)	
Infant asphyxia, *n* (%)	399 (2.1)	691 (2.2)	908 (2.3)	251 (2.9)	124 (2.9)	<0.001
Infant mortality, *n* (%)	125 (0.6)	196 (0.6)	227 (0.6)	58 (0.7)	24 (0.6)	0.52

BMI, body mass index; SD, standard deviation.

a Multinomial logistic regression used for calculation.

**Fig. 1:**
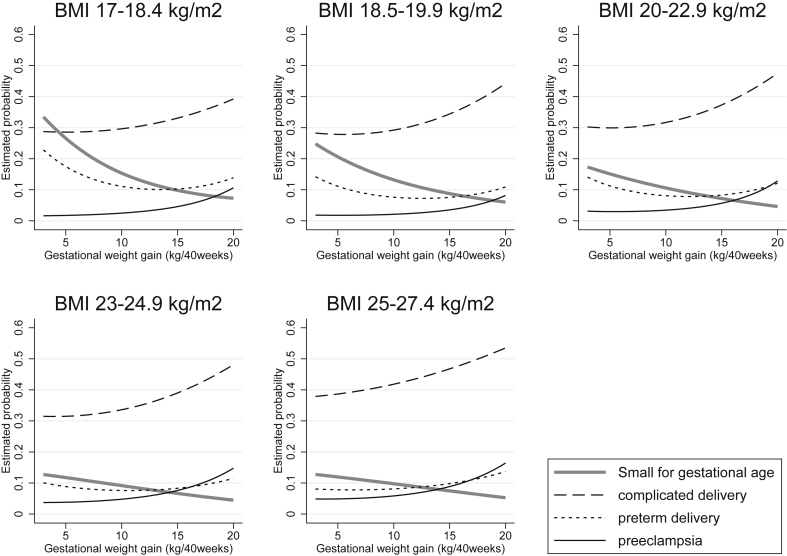
Estimated probability of small for gestational age, preterm delivery, complicated delivery, and preeclampsia, by gestational weight gain rate. Analysis of 104,070 singleton pregnancies in nulliparous Japanese women. The probability is shown for the average non-smoking woman of 160 cm, age 30 years, and mean BMI of each stratum. Complicated delivery includes any of the following: cesarean section, forceps or vacuum delivery, obstructed labor, post-partum hemorrhage. BMI, body mass index; SGA, small for gestational age.

**Fig. 2:**
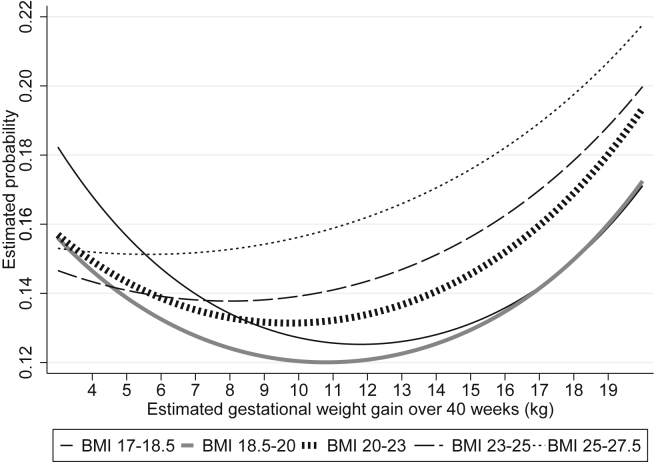
Weighted probability of adverse pregnancy outcomes by gestational weight gain rate, stratified by pre-pregnancy BMI. Analysis of 104,070 singleton pregnancies in nulliparous Japanese women. Complicated delivery includes any of the following: cesarean section, forceps or vacuum delivery, obstructed labor, post-partum hemorrhage. Probability shown is estimated for women of 30 years of age, 160-cm height, and average BMI of each strata (17.9 kg/m^2^, 19.3 kg/m^2^, 21.2 kg/m^2^, 23.8 kg/m^2^ and 26.1 kg/m^2^, respectively). BMI, body mass index.

In Fig. 1, we present the association between gestational weight gain rate and estimated probability of SGA, preterm delivery, and complicated delivery in the parametric model using weight gain rate as a continuous predictor, stratified by pre-pregnancy BMI category. The risk of the combination of adverse outcomes (i.e., weighted combination of SGA, preterm delivery, and complicated delivery) in relation to weight gain is also shown in Fig. 2. For all BMI categories, risk showed a U-shape curve with faster gestational weight gain, which was a result of the tradeoff between decreasing risk of SGA and increasing risk of complicated delivery, and a U-shape curve of risk of preterm delivery. For example, women with BMI 17–18.5 kg/m^2^ who gained weight at the rate of 13 kg over 40 weeks, had 0.75 (95% confidence interval [CI], 0.71–0.78) times less risk of SGA delivery and 0.89 (95% CI, 0.85–0.94) times less risk of preterm delivery, at the cost of 1.18 (95% CI, 1.04–1.12) times greater risk of complicated delivery, compared to those who gained weight at the rate of 10 kg over 40 weeks.

Adjusted relative risk ratios (RRRs) and marginal risk estimates of the risk of adverse pregnancy outcomes according to weight gain rate using the non-parametric model of categorized weight gain rate, after adjusting for maternal background characteristics, are shown in eTable 1 and eFig. 1 for mothers with expected weight gain rate of 3–20 kg over 40 weeks. Similar to the parametric model, faster gestational weight gain was associated with lower risk of SGA, higher risk of LGA, cesarean delivery, complicated delivery, preeclampsia, and severe preeclampsia in all BMI categories. Risk of preterm delivery showed a U-shape curve in BMI categories under 25 kg/m^2^, as well as very preterm birth at BMI 20–22.9 kg/m^2^. For other BMI categories, faster or slower weight gain did not show a distinct association with very preterm delivery.

In Table 2, we show “optimal” rate of gestational weight gain (kg/40 weeks) with lowest risk of adverse outcomes, as well as the “acceptable range” of gestational weight gain defined as an increase in risk compared to optimal weight gain of less than 1%. Optimal rate of weight gain decreased with increasing BMI. When population was limited to women with preeclampsia was excluded from the clinical weights, or the WHO growth chart used instead of the Japanese growth chart, optimal weight gain rate shifted higher for all estimates by 0.2–2.5 kg. However, in all models, the point estimate of optimal gestational weight gain for women of BMI under 18.5 was higher than current Japanese gestational weight gain recommendations, and the point estimate for women of BMI over 23 kg/m^2^ was lower than current IOM recommendations (Table S2). Estimates and optimal gains minimally changed when limited to women with Japanese nationality.

**Table 2:** Estimated gestational weight gain with lowest probability of adverse outcomes, stratified by pre-pregnancy BMI, among 104,070 Japanese nulliparous women.

	BMI (kg/m^2^)				
	17–18.4	18.5–19.9	20–22.9	23–24.9	25–27.4
Optimal and acceptable range of rate of gestational weight gain, kg/40 weeks	12.2 (10.8–13.6)	10.9 (9.5–12.4)	9.9 (8.4–11.4)	7.7 (5.8–9.6)	4.3 (1.7–6.9)
Institute of Medicine Guideline, kg	12.7–18.1	11.3–15.9	11.3–15.9	11.3–15.9	6.8–11.3
Japanese Guideline, kg	9–12	7–12	7–12	7–12	None

BMI, body mass index; SGA, small for gestational age.

## Discussion

### Main findings

We found that the impact of gestational weight gain rate on birth outcomes differed according to pre-pregnancy BMI, to the extent that current Japanese gestational weight gain recommendations may be too strict for women with BMI under 18.5 kg/m^2^ and the IOM recommendations may be too high for Japanese with pre-pregnancy BMI over 23 kg/m^2^. Japanese women with BMI over 23 kg/m^2^ may benefit from being defined as overweight and applying lower gestational weight recommendations compared to women with lower BMI.

### Interpretation

This is the first large study in Japan to estimate optimal gestational weight gain among Japanese women by weighting the importance of multiple outcomes. Determination of the optimal rate of gestational weight gain comes from a trade-off of avoiding adverse events associated with excessive gestational weight gain and those associated with inadequate gestational weight gain. The relative importance of birth outcomes (such as SGA, preterm delivery, cesarean delivery) changes as we learn more of their long-term consequences, and the evolution of the IOM guidelines over time reflects the difficulty in determining the relative importance of these outcomes. Studies on the impact of gestational weight gain on birth outcomes in Asians are scarce compared to those in Caucasians,^33^ with only a few studies stratified by BMI and investigating several birth outcomes concurrently, none of them from Japan.^4^, ^5^, ^18^, ^19^

We found that Japanese women with pre-pregnancy BMI lower than 18.5 kg/m^2^ may have better outcomes if their gestational weight gain exceeds current Japanese recommendations. The JSOG guideline created in 1995 on gestational weight gain had historically been strict (7–10 kg gain for women with BMI of 18–25 kg/m^2^), as prevention of preeclampsia has been of high priority for obstetricians in Japan. The guidelines were revised in 2009 by the Ministry of Health, Labor and Welfare but were still lower than the IOM recommendations. However, even when preeclampsia was considered in our study, all estimates of the optimal gestational weight gain for underweight women exceeded the Japanese guidelines. Therefore, even if higher gestational weight gain should have a causal relationship with increased risk of preeclampsia, the current Japanese recommendations for underweight women are too low.

Many studies have shown that the impact of gestational weight gain rate on birth outcomes largely differs according to pre-pregnancy BMI,^4^, ^5^, ^6^, ^7^, ^16^, ^17^, ^18^ a finding that is consistent with our results. Although a “customized” weight gain chart for specific BMI and height, such as was created in Chile,^34^ may provide a more accurate estimate of optimal weight gain for the individual, its complexity has limited its usability use of simpler BMI-category specific guidelines is more common.^1^, ^2^, ^3^ Thus, recent studies providing optimal weight gain estimates^16^, ^18^ used the broad BMI categories of underweight (<18.5 kg/m^2^), normal BMI (18.5–25 kg/m^2^), overweight (25–30 kg/m^2^) and obese (>30 kg/m^2^).

However, in our study, we re-visited whether these frequently used BMI categories are optimal for women in Japan. Recent studies suggest that Asians have different body composition,^9^ and the American Diabetes Association decided in 2014 to lower the BMI threshold for diabetic screening among Asians from 25 kg/m^2^ to 23 kg/m^2^.^10^, ^11^ Thus, we used narrower BMI categories in our study using the thresholds proposed by WHO, in addition to the traditional thresholds (although it was at the expense of not being able to provide estimates to women with extreme BMI, such as BMI <17.0 kg/m^2^ or ≥27.5 kg/m^2^, which were the lower 4% and upper 3% of the population, respectively), to observe how BMI could be better categorized when recommending weight gain.

Through this approach, we found that Japanese with BMI over 23 kg/m^2^ benefit from lower gestational weight gain than recommended in current IOM guidelines, so direct application of these guidelines to East Asian people should be made with care. Therefore, it may be wise to consider using the WHO-modified BMI threshold to define overweight when applying IOM recommendations to East Asians. Even though the IOM guidelines were not developed for use outside the Unites States or for minority races within the country,^1^ they are currently widely used for many populations that do not have their own national guidelines due to the lack of any other guideline that is based on as much evidence.^2^, ^3^ Most Asian countries do not have a national guideline for gestational weight gain, so they refer to the IOM guidelines for clinical and research use. An increase in Asian immigrants has been observed in many Western countries, including the United States,^35^ Canada,^36^ the United Kingdom,^37^ Australia, and New Zealand,^38^ all of which have primarily adopted the IOM guidelines.

### Strengths and limitations

The strengths of our study include its large sample size, sophisticated statistical methods used to estimate gestational weight gain rate, and reliability of the findings owing to sensitivity analysis using different weights for deciding outcomes.

However, our study has several limitations. First, as we used a birth registry database, we did not have antenatal gestational weight gain measurements for most participants, which has been a limitation in most previous studies as well.^33^ We tried to predict the trajectory of weight gain statistically using total gestational weight gain at delivery; however, this method is hypothetical and could differ from actual measurements. Second, we could only include nulliparous women in our analysis, as we lacked data on previous cesarean delivery. However, as multipara pregnancies are at reduced risk of complicated delivery and preeclampsia, it is likely that optimal gestational weight gain is higher among these women. Third, our database did not include long-term outcomes, such as maternal body weight retention, childhood obesity, or measurements of development, so our analysis was limited to short-term outcomes. Fourth, as our study was conducted among women delivering in Japan only, further research to assess whether findings are generalizable to other Asian populations is needed.

### Conclusion

Our study suggests that current Japanese gestational weight gain guidelines maybe lower than the optimal range for women with BMI below 18.5 kg/m^2^. Optimal weight gain during pregnancy varies largely by pre-pregnancy BMI, and defining those with BMI over 23 kg/m^2^ as overweight, as proposed by the WHO, may be useful if applying current IOM recommendations to women in Japan or when revising Japanese recommendations in the future.

## Conflicts of interest

None declared.

## Disclosures

All authors have nothing to disclose.

## Contribution of authorship

NM initiated the concept and designed the study to which CN, SCJ, EO and FT gave significant advice. SS collected the data, NM analyzed the data and wrote the initial manuscript, which CN SCJ EO SS FT and HS critically reviewed. All authors approved the final manuscript.

## Details of ethical approval

The protocol for this study was approved by the Institutional Review Board of the National Center for Child Health and Development on November 27, 2012 (#2012-9).

## Funding

NM was supported by the Uehara Memorial Foundation Research Grant, Japan Society for the Promotion of Science (KAKENHI 26870889) and the Japan Ministry of Health, Labor and Welfare (H28-ICT-001). NM and SS were supported by the Japan Agency for Medical Research and Development (AMED-6013). EO was supported by the U.S. National Institutes of Health (K24 HD 069408, P30 DK 092924). The funding sources had no involvement in the study design; collection, analysis, or interpretation of data; the writing of the report, nor the decision to submit the article for publication.
