# Supplementary material for: Pre-pregnancy BMI-specific optimal gestational weight gain for women in Japan
Source: J Epidemiol. 2017 May 31;27(10):492–8. doi: 10.1016/j.je.2016.09.013 (PMC5602799; doi:10.1016/j.je.2016.09.013)
Supplement: Supplementary file 1 [file mmc1.pdf]

**eAppendix 1.** Equation to calculate expected gestational weight gain over 40 weeks

To create a model to estimate expected gestational weight gain (GWG) over 40 weeks, we used a subsample of 1283 women who were also participating in a birth cohort and for whom we had detailed measurements from antenatal check-up visits (average 8.2 measurements per woman). For this cohort, pregnant women attending antenatal visits at the National Center for Child Health and Development (NCCHD) from May 13, 2010 until November 28, 2013 were recruited during their first antenatal visit, which usually takes place in weeks 6–14 of gestation. The NCCHD hospital is a tertiary hospital located in suburban Tokyo, and manages approximately 1,500 annual deliveries.

Residual analysis of simple linear regression of gestational length on GWG showed that a linear assumption for our weeks of interest (gestational age 28 weeks to 41 weeks) was adequate. Trajectory analysis, which clustered women into 10 groups by GWG trajectories, showed that there was no significant group of women that failed this assumption. Therefore, we assumed GWG [Y (kg)] could be modeled as a linear factor of gestational length [X (weeks)] and subject-specific GWG speed [A (kg/weeks)] using two constants [x’ (weeks)], [y’ (kg)] universal across subjects.

$$(Y-y') = A(X-x')$$

Using multi-level regression allowing subject specific slopes, we calculated constants to be x’: 2.6 (95% CI; 2.2, 3.1) and y’: -3.8 (95% CI: -3.9, -3.6).

In our population, total GWG was calculated from clinical measurements on admission for delivery and pre-pregnancy weight was ascertained as described above.

**eTable 1.** Estimated relative risk ratios of adverse outcomes by expected gestational weight gain over 40 weeks

**A)** Women with a pre-pregnancy BMI of 17.0–18.4 kg/m$^2$ (analysis of 18,909 women)

| Expected weight gain (kg/40 weeks) | SGA | LGA | Complicated delivery | Cesarean delivery | Preterm delivery | Preeclampsia | Very preterm delivery | Severe preeclampsia |
|---|---|---|---|---|---|---|---|---|
| 3–4.9 | 2.8*** [2.3–3.4] | 0.5* [0.3–0.9] | 1.0 [0.8–1.2] | 1.0 [0.8–1.3] | 2.3*** [1.8–2.9] | 1.2 [0.7–2.0] | 1.5 [0.8–3.0] | 0.9 [0.4–2.1] |
| 5–6.9 | 1.8*** [1.6–2.1] | 0.4*** [0.3–0.6] | 1.0 [0.9–1.1] | 1.0 [0.9–1.2] | 1.7*** [1.5–2.1] | 0.6* [0.4–0.9] | 1.6* [1.0–2.6] | 0.7 [0.4–1.3] |
| 7–7.9 | 1.5*** [1.3–1.8] | 0.4*** [0.2–0.5] | 1.0 [0.9–1.1] | 1.1 [0.9–1.3] | 1.6*** [1.3–1.9] | 0.6* [0.4–0.9] | 1.5 [1.0–2.4] | 0.7 [0.4–1.3] |
| 8–8.9 | 1.5*** [1.3–1.7] | 0.5*** [0.4–0.7] | 1.0 [0.9–1.1] | 0.9 [0.8–1.1] | 1.2* [1.0–1.5] | 0.9 [0.6–1.2] | 0.7 [0.4–1.2] | 1.1 [0.7–1.8] |
| 9–9.9 | 1.2** [1.1–1.4] | 0.7** [0.5–0.9] | 1.0 [0.9–1.1] | 1.0 [0.9–1.2] | 1.1 [0.9–1.3] | 1.0 [0.8–1.4] | 1.1 [0.7–1.8] | 1.3 [0.8–2.1] |
| 10–10.9 | 1 Ref | 1 Ref | 1 Ref | 1 Ref | 1 Ref | 1 Ref | 1 Ref | 1 Ref |
| 11–11.9 | 1.0 [0.8–1.1] | 1.2 [0.9–1.5] | 1.0 [0.9–1.2] | 1.0 [0.9–1.2] | 0.9 [0.7–1.1] | 1.0 [0.7–1.4] | 0.9 [0.6–1.4] | 1.4 [0.9–2.2] |
| 12–12.9 | 0.8** [0.7–0.9] | 1.4** [1.1–1.7] | 1.0 [0.9–1.1] | 1.0 [0.9–1.1] | 0.9 [0.7–1.1] | 1.3 [1.0–1.8] | 0.8 [0.5–1.3] | 1.3 [0.8–2.1] |
| 13–13.9 | 0.8* [0.7–1.0] | 1.4** [1.1–1.8] | 1.1 [1.0–1.2] | 1.1 [1.0–1.3] | 1.1 [0.9–1.4] | 1.6** [1.1–2.1] | 1.1 [0.6–1.7] | 2.1** [1.3–3.3] |
| 14–14.9 | 0.8 [0.7–1.0] | 1.8*** [1.4–2.3] | 1.1 [1.0–1.3] | 1.2* [1.0–1.5] | 0.9 [0.7–1.2] | 2.2*** [1.6–3.0] | 1.2 [0.7–2.1] | 2.7*** [1.7–4.3] |
| 15–15.9 | 0.7** [0.6–0.9] | 1.7*** [1.3–2.3] | 1.4*** [1.2–1.6] | 1.5*** [1.3–1.8] | 1.3* [1.0–1.6] | 2.4*** [1.7–3.3] | 1.8* [1.0–2.9] | 3.4*** [2.1–5.4] |
| 16–16.9 | 0.5*** [0.4–0.7] | 2.6*** [2.0–3.4] | 1.3** [1.1–1.6] | 1.2 [1.0–1.5] | 1.2 [0.9–1.5] | 3.6*** [2.5–5.1] | 1.0 [0.5–2.0] | 5.3*** [3.3–8.5] |
| 17–17.9 | 0.5*** | 2.8*** | 1.3* | 1.2 | 1.0 | 2.6*** | 1.0 | 3.6*** |

| | | | | | | | | |
|---|---|---|---|---|---|---|---|---|
| | [0.3–0.7] | [2.1–3.9] | [1.1–1.6] | [0.9–1.5] | [0.7–1.4] | [1.7–4.1] | [0.4–2.3] | [2.0–6.5] |
| 18–19.9 | 0.6** | 3.2*** | 1.4*** | 1.6*** | 1.1 | 2.9*** | 1.6 | 5.4*** |
| | [0.5–0.9] | [2.3–4.3] | [1.2–1.8] | [1.2–2.0] | [0.8–1.5] | [1.9–4.5] | [0.8–3.2] | [3.2–9.1] |

**B)** Women with a pre-pregnancy BMI of 18.5–19.9 kg/m$^2$ (analysis of 31,286 women)

| Expected weight gain (kg/40 weeks) | SGA | LGA | Complicated delivery | Cesarean delivery | Preterm delivery | Preeclampsia | Very preterm delivery | Severe preeclampsia |
|---|---|---|---|---|---|---|---|---|
| 3–4.9 | 1.8*** | 0.4*** | 1.0 | 1.2 | 2.5*** | 1.2 | 1.3 | 1.1 |
| | [1.5–2.2] | [0.3–0.7] | [0.9–1.2] | [1.0–1.4] | [2.0–3.1] | [0.8–1.8] | [0.7–2.4] | [0.6–2.0] |
| 5–6.9 | 1.5*** | 0.5*** | 1.0 | 1.0 | 1.6*** | 0.8 | 1.6* | 0.8 |
| | [1.3–1.7] | [0.4–0.6] | [0.9–1.1] | [0.9–1.1] | [1.4–1.9] | [0.6–1.1] | [1.1–2.4] | [0.6–1.3] |
| 7–7.9 | 1.5*** | 0.6*** | 0.9 | 0.9 | 1.4*** | 0.9 | 1.5 | 0.9 |
| | [1.3–1.7] | [0.5–0.8] | [0.8–1.0] | [0.8–1.1] | [1.2–1.7] | [0.7–1.2] | [1.0–2.3] | [0.6–1.4] |
| 8–8.9 | 1.2* | 0.7*** | 1.0 | 0.9 | 1.2* | 0.8 | 1.1 | 0.7 |
| | [1.0–1.3] | [0.6–0.9] | [0.9–1.1] | [0.8–1.1] | [1.0–1.5] | [0.6–1.0] | [0.7–1.6] | [0.5–1.0] |
| 9–9.9 | 1.0 | 1.0 | 1.0 | 1.0 | 1.2* | 0.8* | 0.9 | 0.7 |
| | [0.9–1.1] | [0.8–1.2] | [0.9–1.1] | [0.9–1.1] | [1.0–1.4] | [0.6–1.0] | [0.6–1.3] | [0.5–1.1] |
| 10–10.9 | 1 | 1 | 1 | 1 | 1 | 1 | 1 | 1 |
| | Ref | Ref | Ref | Ref | Ref | Ref | Ref | Ref |
| 11–11.9 | 0.9 | 1.2 | 1.1** | 1.1 | 1.1 | 1.0 | 0.9 | 1.1 |
| | [0.8–1.0] | [1.0–1.4] | [1.0–1.3] | [1.0–1.2] | [1.0–1.3] | [0.8–1.3] | [0.6–1.4] | [0.8–1.5] |
| 12–12.9 | 0.8** | 1.4*** | 1.2** | 1.2** | 1.4*** | 1.4* | 1.1 | 1.7** |
| | [0.7–0.9] | [1.2–1.7] | [1.1–1.3] | [1.1–1.3] | [1.2–1.6] | [1.1–1.7] | [0.8–1.7] | [1.2–2.4] |
| 13–13.9 | 0.7*** | 1.5*** | 1.2** | 1.2** | 1.3** | 1.5** | 1.0 | 1.9*** |
| | [0.6–0.8] | [1.3–1.8] | [1.1–1.3] | [1.1–1.4] | [1.1–1.5] | [1.2–1.9] | [0.6–1.5] | [1.3–2.7] |
| 14–14.9 | 0.6*** | 1.7*** | 1.2* | 1.2* | 1.2 | 1.6*** | 1.7* | 1.9** |
| | [0.5–0.8] | [1.4–2.0] | [1.0–1.3] | [1.0–1.4] | [1.0–1.4] | [1.2–2.2] | [1.1–2.7] | [1.3–2.8] |
| 15–15.9 | 0.7** | 1.9*** | 1.4*** | 1.5*** | 1.4** | 2.0*** | 1.4 | 2.4*** |
| | [0.6–0.9] | [1.6–2.4] | [1.3–1.6] | [1.3–1.7] | [1.1–1.7] | [1.5–2.7] | [0.8–2.3] | [1.6–3.5] |

| | | | | | | | | |
|---|---|---|---|---|---|---|---|---|
| 16–16.9 | 0.6*** [0.5–0.8] | 2.2*** [1.7–2.7] | 1.5*** [1.3–1.7] | 1.5*** [1.3–1.8] | 1.4* [1.1–1.7] | 2.0*** [1.4–2.7] | 0.7 [0.3–1.5] | 2.3*** [1.5–3.6] |
| 17–17.9 | 0.5*** [0.4–0.7] | 2.4*** [1.8–3.0] | 1.8*** [1.5–2.2] | 1.9*** [1.5–2.3] | 1.1 [0.8–1.5] | 2.2*** [1.5–3.2] | 1.6 [0.8–3.0] | 2.6*** [1.6–4.2] |
| 18–19.9 | 0.6** [0.4–0.8] | 3.1*** [2.5–3.9] | 1.6*** [1.3–1.9] | 1.8*** [1.5–2.2] | 1.2 [0.9–1.6] | 2.6*** [1.8–3.7] | 1.8 [1.0–3.3] | 3.3*** [2.1–5.2] |

**C)** Women with a pre-pregnancy BMI of 20.0–22.9 kg/m$^2$ (analysis of 38,222 women)

| Expected weight gain (kg/40 weeks) | SGA | LGA | Complicated delivery | Cesarean delivery | Preterm delivery | Preeclampsia | Very preterm delivery | Severe preeclampsia |
|---|---|---|---|---|---|---|---|---|
| 3–4.9 | 1.8*** [1.5–2.1] | 0.6*** [0.5–0.8] | 0.9 [0.8–1.1] | 1.1 [0.9–1.2] | 2.1*** [1.7–2.5] | 0.9 [0.7–1.2] | 2.5*** [1.7–3.8] | 0.9 [0.6–1.4] |
| 5–6.9 | 1.5*** [1.3–1.7] | 0.7*** [0.6–0.8] | 0.9 [0.8–1.0] | 1.0 [0.9–1.1] | 1.4*** [1.2–1.6] | 0.8* [0.6–1.0] | 2.0*** [1.4–2.8] | 0.8 [0.6–1.1] |
| 7–7.9 | 1.4*** [1.2–1.6] | 0.7*** [0.6–0.9] | 1.0 [0.9–1.1] | 1.0 [0.9–1.1] | 1.3** [1.1–1.5] | 0.9 [0.7–1.1] | 1.4 [1.0–2.1] | 1.0 [0.7–1.3] |
| 8–8.9 | 1.2** [1.1–1.4] | 0.7*** [0.6–0.8] | 1.0 [0.9–1.1] | 0.9 [0.8–1.0] | 1.1 [1.0–1.3] | 0.9 [0.7–1.1] | 1.2 [0.8–1.8] | 0.8 [0.6–1.1] |
| 9–9.9 | 1.2** [1.1–1.4] | 0.9 [0.8–1.1] | 1.0 [0.9–1.1] | 1.0 [0.9–1.1] | 1.2* [1.0–1.4] | 0.8 [0.7–1.0] | 1.3 [0.9–1.8] | 0.8 [0.6–1.1] |
| 10–10.9 | 1 Ref | 1 Ref | 1 Ref | 1 Ref | 1 Ref | 1 Ref | 1 Ref | 1 Ref |
| 11–11.9 | 1.1 [0.9–1.2] | 1.1 [1.0–1.3] | 1.1** [1.0–1.2] | 1.1* [1.0–1.2] | 1.1 [1.0–1.3] | 1.0 [0.8–1.2] | 1.1 [0.8–1.6] | 1.2 [0.9–1.6] |
| 12–12.9 | 0.9 [0.8–1.0] | 1.4*** [1.2–1.6] | 1.2*** [1.1–1.3] | 1.2** [1.1–1.3] | 1.0 [0.9–1.2] | 1.1 [0.9–1.3] | 0.9 [0.6–1.3] | 1.2 [0.9–1.6] |
| 13–13.9 | 0.9 [0.8–1.0] | 1.5*** [1.3–1.7] | 1.2*** [1.1–1.3] | 1.2** [1.1–1.3] | 1.0 [0.8–1.2] | 1.3** [1.1–1.6] | 0.9 [0.6–1.4] | 1.7*** [1.3–2.2] |

| | | | | | | | | |
|---|---|---|---|---|---|---|---|---|
| 14–14.9 | 0.7*** [0.6–0.8] | 1.7*** [1.5–2.0] | 1.2*** [1.1–1.4] | 1.2* [1.0–1.3] | 1.0 [0.8–1.2] | 1.4** [1.1–1.8] | 1.2 [0.8–1.9] | 1.6** [1.1–2.2] |
| 15–15.9 | 0.8* [0.6–1.0] | 1.7*** [1.4–2.0] | 1.3*** [1.2–1.5] | 1.4*** [1.3–1.6] | 1.5*** [1.2–1.7] | 2.0*** [1.6–2.5] | 1.7* [1.1–2.6] | 2.3*** [1.7–3.1] |
| 16–16.9 | 0.7** [0.6–0.9] | 1.8*** [1.5–2.2] | 1.4*** [1.2–1.6] | 1.6*** [1.4–1.9] | 1.2 [1.0–1.5] | 2.3*** [1.8–2.9] | 2.0** [1.2–3.1] | 2.5*** [1.8–3.5] |
| 17–17.9 | 0.6** [0.5–0.9] | 2.4*** [2.0–3.0] | 1.4*** [1.2–1.7] | 1.6*** [1.4–1.9] | 1.4** [1.1–1.8] | 2.3*** [1.8–3.1] | 2.2** [1.3–3.6] | 2.4*** [1.6–3.6] |
| 18–19.9 | 0.6*** [0.4–0.8] | 2.5*** [2.1–3.0] | 2.0*** [1.8–2.4] | 2.2*** [1.9–2.6] | 1.5*** [1.2–1.9] | 3.3*** [2.6–4.1] | 2.3*** [1.5–3.8] | 3.7*** [2.7–5.1] |

**D)** Women with a pre-pregnancy BMI of 23–24.9 kg/m$^2$ (analysis of 8,159 women)

| Expected weight gain (kg/40 weeks) | SGA | LGA | Complicated delivery | Cesarean delivery | Preterm delivery | Preeclampsia | Very preterm delivery | Severe preeclampsia |
|---|---|---|---|---|---|---|---|---|
| 3–4.9 | 1.6** [1.2–2.3] | 0.6** [0.4–0.9] | 0.9 [0.7–1.1] | 1.1 [0.8–1.4] | 1.2 [0.8–1.7] | 0.7 [0.5–1.1] | 1.0 [0.5–2.2] | 0.6 [0.3–1.2] |
| 5–6.9 | 1.4* [1.0–1.8] | 0.5*** [0.4–0.7] | 1.0 [0.9–1.3] | 1.0 [0.8–1.3] | 1.2 [0.9–1.6] | 0.8 [0.6–1.2] | 1.5 [0.8–2.7] | 0.7 [0.4–1.3] |
| 7–7.9 | 1.1 [0.8–1.5] | 0.6** [0.5–0.9] | 0.9 [0.8–1.2] | 1.2 [0.9–1.5] | 1.1 [0.8–1.5] | 0.7 [0.5–1.0] | 0.8 [0.4–1.7] | 0.6 [0.3–1.1] |
| 8–8.9 | 1.0 [0.7–1.3] | 0.8 [0.6–1.1] | 1.0 [0.8–1.3] | 1.0 [0.8–1.3] | 1.0 [0.7–1.4] | 0.7 [0.5–1.0] | 0.8 [0.4–1.7] | 0.7 [0.4–1.3] |
| 9–9.9 | 1.1 [0.8–1.5] | 0.8 [0.6–1.1] | 1.1 [0.9–1.3] | 1.2 [0.9–1.5] | 0.8 [0.6–1.2] | 0.8 [0.6–1.1] | 0.9 [0.5–1.8] | 1.1 [0.7–1.8] |
| 10–10.9 | 1 Ref | 1 Ref | 1 Ref | 1 Ref | 1 Ref | 1 Ref | 1 Ref | 1 Ref |
| 11–11.9 | 0.9 [0.7–1.3] | 1.0 [0.7–1.3] | 1.2 [1.0–1.4] | 1.2 [1.0–1.5] | 0.8 [0.6–1.2] | 0.8 [0.5–1.1] | 0.8 [0.4–1.7] | 0.7 [0.4–1.2] |
| 12–12.9 | 1.0 | 1.1 | 1.2 | 1.3* | 0.9 | 1.1 | 0.7 | 1.1 |

| | | | | | | | | |
|---|---|---|---|---|---|---|---|---|
| | [0.7–1.4] | [0.8–1.5] | [1.0–1.4] | [1.1–1.7] | [0.6–1.2] | [0.7–1.5] | [0.3–1.5] | [0.7–1.9] |
| 13–13.9 | 1.0 | 1.2 | 1.4** | 1.6*** | 1.3 | 1.6* | 1.4 | 1.6 |
| | [0.7–1.4] | [0.9–1.7] | [1.1–1.7] | [1.2–2.0] | [1.0–1.9] | [1.1–2.3] | [0.7–2.8] | [1.0–2.7] |
| 14–14.9 | 1.0 | 1.3 | 1.3* | 1.4* | 0.8 | 1.4 | 0.9 | 1.2 |
| | [0.7–1.5] | [1.0–1.9] | [1.0–1.7] | [1.1–1.8] | [0.5–1.2] | [0.9–2.1] | [0.4–2.1] | [0.6–2.1] |
| 15–15.9 | 0.8 | 1.8*** | 1.5*** | 1.4* | 0.9 | 1.5 | 1.2 | 1.4 |
| | [0.5–1.2] | [1.3–2.5] | [1.2–1.9] | [1.1–1.9] | [0.6–1.4] | [1.0–2.2] | [0.5–2.7] | [0.7–2.5] |
| 16–16.9 | 0.6 | 1.8** | 1.5* | 1.9*** | 1.4 | 1.6* | 0.6 | 1.9 |
| | [0.3–1.1] | [1.3–2.6] | [1.1–1.9] | [1.4–2.6] | [0.9–2.2] | [1.0–2.6] | [0.2–2.2] | [1.0–3.5] |
| 17–17.9 | 0.3** | 1.4 | 1.5* | 2.0*** | 1.6* | 2.3*** | 1.3 | 2.5** |
| | [0.1–0.7] | [0.9–2.0] | [1.1–2.0] | [1.4–2.8] | [1.0–2.5] | [1.5–3.6] | [0.5–3.5] | [1.3–4.6] |
| 18–19.9 | 0.6 | 1.6* | 1.9*** | 2.4*** | 1.7* | 2.0** | 1.4 | 2.1* |
| | [0.3–1.1] | [1.1–2.3] | [1.4–2.5] | [1.8–3.3] | [1.1–2.6] | [1.3–3.1] | [0.6–3.7] | [1.1–3.9] |

E) Women with a pre-pregnancy BMI of 25–27.4 kg/m$^2$ (analysis of 3,843 women)

| Expected weight gain (kg/40 weeks) | SGA | LGA | Complicated delivery | Cesarean delivery | Preterm delivery | Preeclampsia | Very preterm delivery | Severe preeclampsia |
|---|---|---|---|---|---|---|---|---|
| 3–4.9 | 0.4 | 0.8 | 0.5*** | 0.7* | 0.7* | 1.3 | 0.9 | 1.2 |
| | [0.1–1.4] | [0.5–1.4] | [0.3–0.7] | [0.5–0.9] | [0.5–1.0] | [0.8–2.1] | [0.5–1.5] | [0.4–3.3] |
| 5–6.9 | 0.2* | 1.1 | 0.5*** | 0.9 | 0.9 | 1.0 | 0.8 | 0.8 |
| | [0.0–0.8] | [0.7–1.7] | [0.4–0.8] | [0.7–1.1] | [0.7–1.2] | [0.6–1.5] | [0.5–1.3] | [0.3–2.2] |
| 7–7.9 | 0.5 | 1.1 | 0.7* | 0.8 | 0.8 | 0.9 | 1.0 | 0.9 |
| | [0.2–1.7] | [0.7–1.7] | [0.4–1.0] | [0.6–1.1] | [0.5–1.0] | [0.6–1.5] | [0.6–1.7] | [0.3–2.6] |
| 8–8.9 | 0.6 | 1.3 | 0.5** | 1.0 | 0.8 | 1.0 | 1.2 | 0.8 |
| | [0.2–1.9] | [0.8–2.0] | [0.3–0.8] | [0.7–1.3] | [0.6–1.1] | [0.6–1.6] | [0.7–2.0] | [0.3–2.4] |
| 9–9.9 | 0.6 | 0.9 | 0.7 | 0.8 | 0.8 | 1.5 | 1.1 | 1.3 |
| | [0.2–1.9] | [0.6–1.5] | [0.4–1.0] | [0.6–1.1] | [0.6–1.2] | [1.0–2.4] | [0.7–1.8] | [0.5–3.5] |
| 10–10.9 | 1 | 1 | 1 | 1 | 1 | 1 | 1 | 1 |
| | Ref | Ref | Ref | Ref | Ref | Ref | Ref | Ref |

| | | | | | | | | |
|---|---|---|---|---|---|---|---|---|
| 11–11.9 | 1.1 [0.4–2.9] | 1.1 [0.7–1.8] | 0.8 [0.6–1.3] | 0.9 [0.6–1.2] | 0.9 [0.6–1.2] | 0.9 [0.5–1.5] | 1.2 [0.7–2.0] | 0.5 [0.1–1.8] |
| 12–12.9 | 0.7 [0.2–2.4] | 0.6 [0.4–1.2] | 1.0 [0.7–1.5] | 1.0 [0.8–1.4] | 1.2 [0.9–1.7] | 1.9** [1.2–3.0] | 1.5 [0.9–2.5] | 1.8 [0.7–4.8] |
| 13–13.9 | 0.8 [0.3–2.8] | 0.7 [0.4–1.4] | 1.0 [0.6–1.5] | 0.9 [0.6–1.2] | 1.1 [0.8–1.6] | 1.0 [0.6–1.8] | 1.7* [1.0–2.9] | 0.7 [0.2–2.8] |
| 14–14.9 | 0.0 [0.0 –0.0] | 0.3* [0.1–0.7] | 1.1 [0.7–1.8] | 1.1 [0.8–1.7] | 1.3 [0.9–2.0] | 0.8 [0.4–1.6] | 1.3 [0.7–2.5] | 1.7 [0.6–5.5] |
| 15–15.9 | 1.5 [0.4–5.6] | 1.1 [0.6–2.0] | 0.8 [0.5–1.4] | 1.2 [0.8–1.8] | 1.4 [0.9–2.1] | 2.4** [1.4–4.2] | 2.3** [1.3–4.2] | 1.1 [0.3–4.2] |
| 16–16.9 | 1.1 [0.2–5.1] | 1.4 [0.7–2.8] | 1.6 [1.0–2.7] | 1.0 [0.6–1.6] | 1.1 [0.7–1.8] | 1.8 [1.0–3.4] | 1.8 [0.9–3.5] | 1.5 [0.4–5.7] |
| 17–17.9 | 1.9 [0.5–7.2] | 0.8 [0.3–1.9] | 1.1 [0.6–2.0] | 1.4 [0.8–2.2] | 1.5 [0.9–2.6] | 1.1 [0.5–2.6] | 2.1* [1.0–4.2] | NE |
| 18–19.9 | 4.6** [1.6–13.1 ] | 0.2* [0.1–0.9] | 1.4 [0.8–2.5] | 1.5 [1.0–2.4] | 1.7* [1.0–2.7] | 1.5 [0.7–3.0] | 2.7** [1.4–5.1] | 1.1 [0.2–5.3] |

BMI, body mass index; SGA, small for gestational age; LGA, large for gestational age; NE, not estimable.

* $p<0.05$; ** $p<0.01$; *** $p<0.001$

Complicated delivery includes any of the following: cesarean section, mechanical delivery, obstructed labor, post-partum hemorrhage.

**eTable 2.** Sensitivity analysis of expected gestational weight gain over 40 weeks with lowest probability of adverse outcomes, stratified by pre-pregnancy BMI

| | | BMI (kg/m$^2$) | | | | |
|---|---|---|---|---|---|---|
| | | 17–18.4 | 18.5–19.9 | 20–22.9 | 23–24.9 | 25–27.4 |
| | | Optimal gestational weight gain over 40 weeks (kg) | | | | |
| Using Japanese definition of SGA | Limited population to women with 3–20 kg of weight gain | 12.5 (10.5–14.4) | 11.2 (9.4–13.1) | 10.4 (8.4–12.4) | 9.3 (7.3–11.5) | 4.5 (NE–7.7) |
| | Least weighted probability of outcomes excluding preeclampsia | 13.1 (11.2–14.8) | 11.7 (10.3–13.0) | 11.0 (9.6–12.4) | 9.8 (8.7–11.0) | 4.6 (NE–7.5) |
| Using WHO definition of SGA | Least weighted probability of adverse outcomes | 13.3 (11.4–15.1) | 12.1 (10.4–13.8) | 11.1 (9.1–13.0) | 9.7 (7.7–11.8) | 4.9 (NE–8.3) |
| | Least weighted probability of outcomes excluding preeclampsia | 14.1 (12.6–15.7) | 12.7 (11.2–14.2) | 11.8 (9.7–13.8) | 10.3 (8.1–12.5) | 5.3 (NE–9.4) |
| Institute of Medicine Guideline (kg) | | 12.7–18.1 | 11.3–15.9 | 11.3–15.9 | 11.3–15.9 | 6.8–11.3 |
| Japanese Guideline (kg) | | 9–12 | 7–12 | 7–12 | 7–12 | none |

BMI, body mass index; SGA, small for gestational age; WHO, World Health Organization.

Complicated delivery includes any of the following: cesarean section, mechanical delivery, obstructed labor, post-partum hemorrhage. For the main analysis, four outcomes (SGA, preterm delivery, complicated delivery, preeclampsia) were weighted by clinical relevance, which was calculated from obstetrician and neonatologist answers as follows: preterm delivery 1.8, preeclampsia 1.7 complicated delivery 1.35, SGA 1.

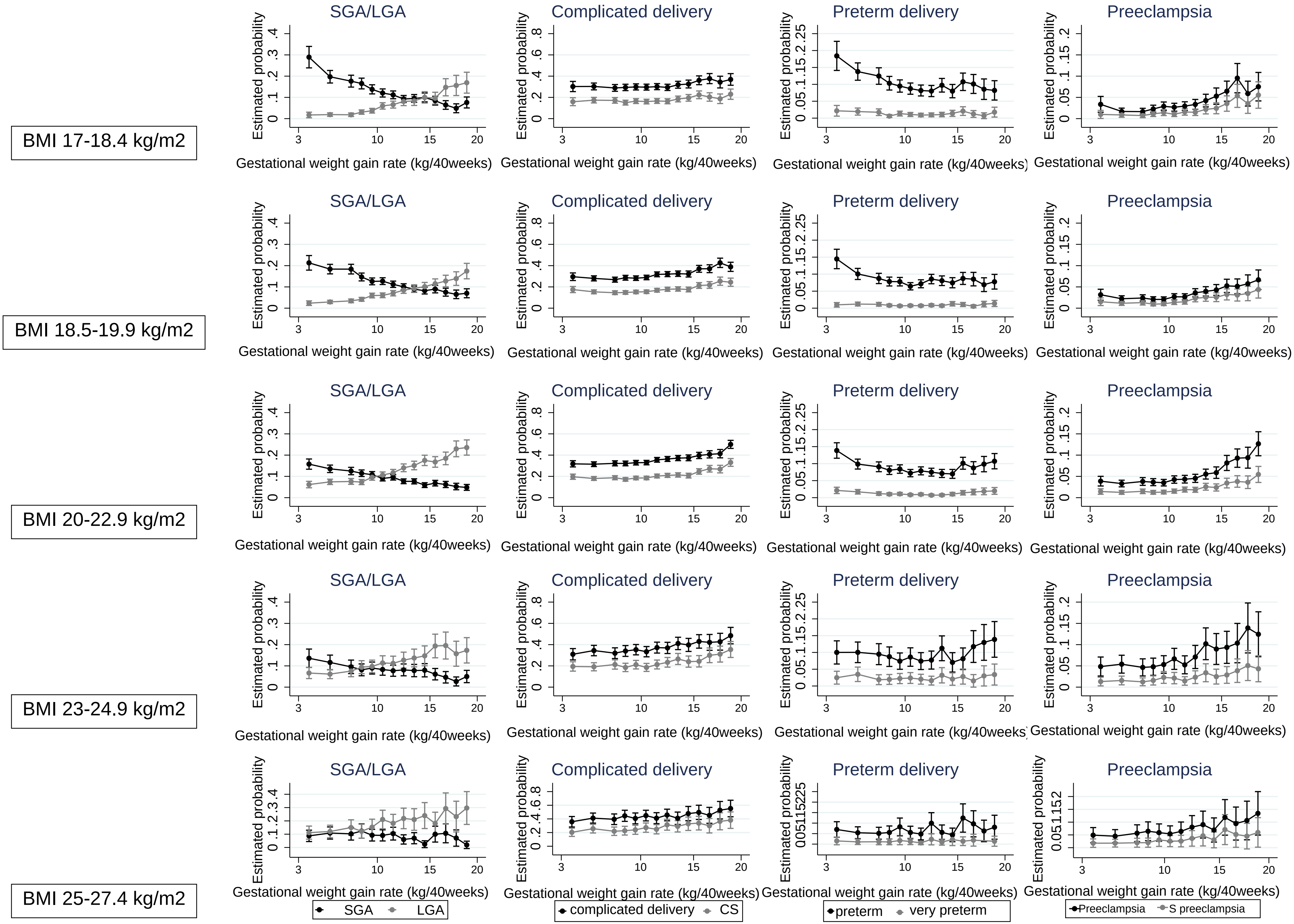
BMI 17-18.4 kg/m2
BMI 18.5-19.9 kg/m2
BMI 20-22.9 kg/m2
BMI 23-24.9 kg/m2
BMI 25-27.4 kg/m2
SGA/LGA
Complicated delivery
Preterm delivery
Preeclampsia
Estimated probability
Gestational weight gain rate (kg/40weeks)
SGA
LGA
complicated delivery
CS
preterm
very preterm
Preeclampsia
S preeclampsia
